# TRPA1 Regulates Fibrosis‐Associated Transcriptional Pathways in Human Lung Epithelial Cells

**DOI:** 10.1111/bcpt.70276

**Published:** 2026-08-05

**Authors:** Leevi Halonen, Samu Luostarinen, Ida Valjus, Julia Vistbacka, Antti Pemmari, Eeva Moilanen

**Affiliations:** ^1^ The Immunopharmacology Research Group, Faculty of Medicine and Health Technology, Tampere University; and Tampere University Hospital, Wellbeing Services County of Pirkanmaa Tampere Finland

## Abstract

Transient receptor potential ankyrin 1 (TRPA1) is a cation channel originally identified in lung fibroblasts and extensively studied in sensory neurons, where it is associated with pain and neurogenic inflammation. We and others have recently shown that TRPA1 is also expressed in lung epithelial cells and that its expression is regulated by the cytokine environment. In the present study, we used next‐generation sequencing to analyse the role of TRPA1 and the effects of its inhibition on human A549 lung epithelial cell phenotype under inflammatory conditions. Two different TRPA1 inhibitors (HC‐030031 and A‐967079) were used and found to alter the expression of 968 genes: 576 genes were downregulated, and 392 upregulated. Ingenuity Pathway Analysis (IPA) predicted that TRPA1 inhibitors significantly suppress the activity of *pulmonary fibrosis idiopathic signalling* and *wound healing* pathways. In these pathways, altered genes included matrix metalloproteinases (MMPs), growth factors, collagens and transcription factors. The major profibrotic mediators MMP‐12 and MMP‐7 were among the most strongly inhibited genes by TRPA1 inhibitors. STRING network analysis suggests that TRPA1 regulates fibrosis‐related genes through FOS gene and AP‐1 transcription factor. These findings together with previous data propose TRPA1 as a factor and therapeutic target in fibrotic lung diseases, which are associated with poor survival and critical need for novel disease‐modifying therapies.

## Summary

- TRPA1 inhibitors were found to regulate the expression of > 900 genes in lung epithelial cells under inflammation.
- Ingenuity Pathway Analysis (IPA) predicted that TRPA1 blockers decreased the activity of pulmonary fibrosis–related pathways.
- MMP‐12 and MMP‐7 were upregulated by inflammatory cytokines in a TRPA1‐dependent manner.
- TRPA1 is a promising therapeutic target for fibrotic and inflammatory lung diseases.

## Plain Language Summary

TRPA1 is an ion channel originally identified in lung fibroblasts and extensively studied in sensory neurons. We and others have recently shown that TRPA1 is also expressed in lung epithelial cells under inflammatory conditions. Here, we investigated the role of TRPA1 and its inhibition in shaping lung epithelial cell phenotype using RNA‐seq and pathway analysis. TRPA1 antagonists altered the expression of > 900 genes and suppressed pathways associated with the development of pulmonary fibrosis. These findings highlight TRPA1 as a promising therapeutic target for fibrotic lung diseases, which are associated with poor survival and critical unmet needs for disease‐modifying therapies.

## Graphical abstract

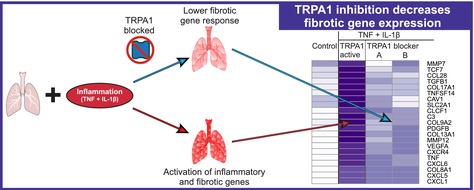

## 1 Introduction

Transient receptor potential ankyrin 1 (TRPA1) belongs to the transient receptor potential (TRP) superfamily of ion channels. It is the only member of the ankyrin family of TRP channels and non‐selectively permeates cations, especially Ca^2+^ and Na^+^. TRPA1 was originally found in lung fibroblasts and named ankyrin‐like protein with transmembrane proteins 1 (ANKTM1) [1]. Early research on TRPA1 focused on the channel's role in neurons as a possible sensor for cold temperatures [2]—a theory that has been controversial since then. TRPA1 was soon found to be activated by exogenous pungent compounds such as cinnamaldehyde (found in cinnamon) [3], allyl isothiocyanate (AITC, found in mustard oil) and allicin (found in garlic) [4]. Later studies have shown that TRPA1 is also expressed in some nonneuronal cells such as lung epithelial cells [5, 6], chondrocytes [7] and keratinocytes [8, 9].

Structurally, TRPA1 is a homotetramer, with each subunit consisting of six helical transmembrane domains S1–S6, and intracellular C and N termini. The intracellular N‐terminal contains ankyrin repeat domain (ARD) with 14–18 characteristic ankyrin repeats and a pre‐S1 region that contains cysteine residues that are target sites for electrophilic TRPA1 agonists, such as AITC and cinnamaldehyde [10]. In addition to electrophilic agonists acting on the pre‐S1 region, TRPA1 seems to be gated by Ca^2+^ [11]. TRPA1 activity is also regulated by G‐protein‐coupled receptor (GPCR) signalling, for example, through phospholipase C (PLC) [3, 12, 13], protein kinase A (PKA) [13] and Gγβ signalling [12, 14].

The expression and function of TRPA1 seem to be modulated by endogenous inflammatory mediators. Pro‐inflammatory cytokines such as interleukin 1β (IL‐1β) and tumour necrosis factor (TNF) increase the expression of TRPA1 [5, 7, 15], whereas interleukin 1α (IL‐1α) has been shown to increase the localization of TRPA1 to the cell membrane [16]. TRPA1 is activated and sensitized by bradykinin [3, 17], a bronchoconstrictive pro‐inflammatory mediator secreted during acute inflammation, allergic reactions and asthma [18]. In addition, TRPA1 is activated by reactive oxygen (ROS) and nitrogen (RNS) species produced in higher amounts in oxidative stress and inflammatory conditions [19, 20].

Recent research implies that TRPA1 may have a role in airway inflammation. Cigarette smoke upregulates TRPA1 expression in alveolar macrophages [21] and activates TRPA1 through its compounds such as acrolein [22] or through production of ROS [23]. TRPA1 activity has also been linked to airway hyperreactivity [24]. We have previously shown that pro‐inflammatory cytokines TNF and IL‐1β increase the expression of TRPA1 in lung epithelial cells [5] and that the expression is further regulated by Th1 and Th2 cytokines [25]. Thus, the expression and activity of TRPA1 are likely enhanced in lung inflammation, but its consequences in lung epithelium remain poorly known. In the present study, we used next‐generation sequencing to analyse transcriptomic changes in human A549 lung epithelial cells, aiming to elucidate the role of TRPA1 and the effects of TRPA1 antagonists on epithelial cell phenotype in the context of inflammatory lung pathophysiology.

## 2 Methods

### 2.1 Cell Culture

Human A549 lung epithelial cells (American Type Culture Collection, Manassas, VA, USA) were cultured in Ham's F‐12K (Kaighn's modification) medium supplemented with 10% heat‐inactivated fetal bovine serum, 100 μg/mL of streptomycin, 100 U/mL of penicillin and 250 ng/mL of amphotericin B (all from Gibco/Life Technologies, Carlsbad, CA, USA) at 37°C in 5% CO_2_. The experiments were carried out using cells at passages six to sixteen. No passage‐dependent differences in cellular responses were observed. The cells were seeded on 24‐well plates and grown for 48 h before the experiments were started. During the experiments, the cells were cultured with the following compounds or their combinations indicated as follows: the pro‐inflammatory cytokines TNFα and IL‐1β (R&D Systems Europe Ltd., Abingdon, UK), the TRPA1 antagonists HC‐030031 or A‐967079 (Sigma‐Aldrich, St. Louis, MO, USA), the glucocorticoid fluticasone propionate (Sigma‐Aldrich), the JAK inhibitors baricitinib or tofacitinib (Selleck Biotechnology GmBH, Cologne, Germany) or the antifibrotic drug nintedanib (Sigma‐Aldrich). The concentrations used were based on our previous studies [5, 25] and preliminary experiments.

### 2.2 Next‐Generation RNA Sequencing

Total RNA was extracted after culturing the cells with compounds of interest for 24 h using GenElute Mammalian Total RNA Miniprep kit (Sigma‐Aldrich) with the addition of On‐Column DNase I Digestion Set (Sigma‐Aldrich). RNA concentration was measured and integrity confirmed with the 2100 Bioanalyzer (Agilent Technologies). Human RNA sequencing was performed by Novogene (Beijing, China) using Illumina sequencing (*n* = 4). Read alignment to reference genome (GRCh38/hg38) was done with Hisat2 v2.0.5 [26], and mapped reads were counted with featureCounts v1.5.0‐p3 [27]. Differential expression analysis was conducted with the DESeq2R package [28]. Functional analysis was performed using Ingenuity Pathway Analysis (IPA) by Qiagen. Protein–protein interactions were analysed with STRING [29].

### 2.3 Reverse Transcriptase Polymerase Chain Reaction (RT‐PCR)

Total RNA was extracted after culturing the cells with compounds of interest for 24 h using GenElute Mammalian Total RNA Miniprep kit (Sigma‐Aldrich) and reverse‐transcribed with Maxima First Strand cDNA Synthesis Kit for RT‐PCR (Thermo Fisher Scientific, Waltham, MA, USA). PCR was conducted with Applied Biosystems 7500 Real‐Time PCR instrument using TaqMan Universal PCR Master Mix and TaqMan Gene Expression assays (Thermo Fisher Scientific) for RPL‐30 (Hs00265497_m1), TRPA1 (Hs00175798_m1), MMP‐7 (Hs01042796_m1) and MMP‐12 (Hs00159178_m1). RT‐PCR results were analysed using the ΔΔCt method. RPL‐30 was chosen for reference gene [30], as our experiments showed it has stable Ct levels across all treatments. For statistical analysis, ΔΔCt values were used. For visual clarity, mean ΔΔCt and SEM were transformed into fold change (2^−ΔΔCt^) in figures.

### 2.4 Immunoassay

Enzyme‐linked immunosorbent assay (ELISA) was used to measure concentrations of MMP‐12 and MMP‐7 in A549 culture medium. Invitrogen Human MMP‐12 ELISA Kit (Thermo Fisher Scientific) and Human Total MMP‐7 DuoSet ELISA (R&D Systems Europe Ltd.) were used in the measurements.

### 2.5 Statistical Analysis and Figures

Statistical comparisons were conducted with Welch ANOVA followed by Holm–Bonferroni corrected groupwise comparisons if ANOVA showed statistically significant difference. The *p* values less than 0.05 were considered statistically significant. For RNA sequencing, the *p* values from differential expression analysis were false discovery rate (FDR) corrected with the Benjamini–Hochberg method. The heatmap was created with variance stabilized and normalized counts. The R software version 4.5.1 [31] was used for statistical analysis with following packages used for data analysis or visualization: dplyr [32], DESeq2 [28], pheatmap [33] and RColorBrewer [34]. Bar graphs were created using GraphPad Prism 10.0.1 for Windows (GraphPad Software, Boston, MA, USA).

The study was conducted in accordance with the *Basic & Clinical Pharmacology & Toxicology* policy for experimental and clinical studies [35].

## 3 Results

### 3.1 Differentially Expressed Genes in Lung Epithelial Cells

In A549 lung epithelial cells treated with TNF and IL‐1β, a total of 3462 genes were significantly upregulated and 3089 were downregulated after normalization and correction for multiple testing (significant change in expression: |FC| > 1.5 and FDR < 0.05). The expression of TRPA1 was increased to 2.64‐fold (*p* < 10^−6^) by TNF + IL‐1β in RNA‐seq analysis, which also repeated in RT‐PCR measurements.

The effect of TRPA1 was assessed by treating the A549 cells with the TRPA1 antagonist A‐967079 or HC‐030031, and genes affected by both antagonists (|FC| > 1.5 and FDR < 0.05) were included in the analysis. When comparing cells treated with the combination of TNF, IL‐1β and TRPA1 antagonist to cells treated with TNF and IL‐1β only, both TRPA1 antagonists significantly upregulated a total of 392 genes and downregulated 576 genes.

### 3.2 Pathway Analysis

**FIGURE 1:**
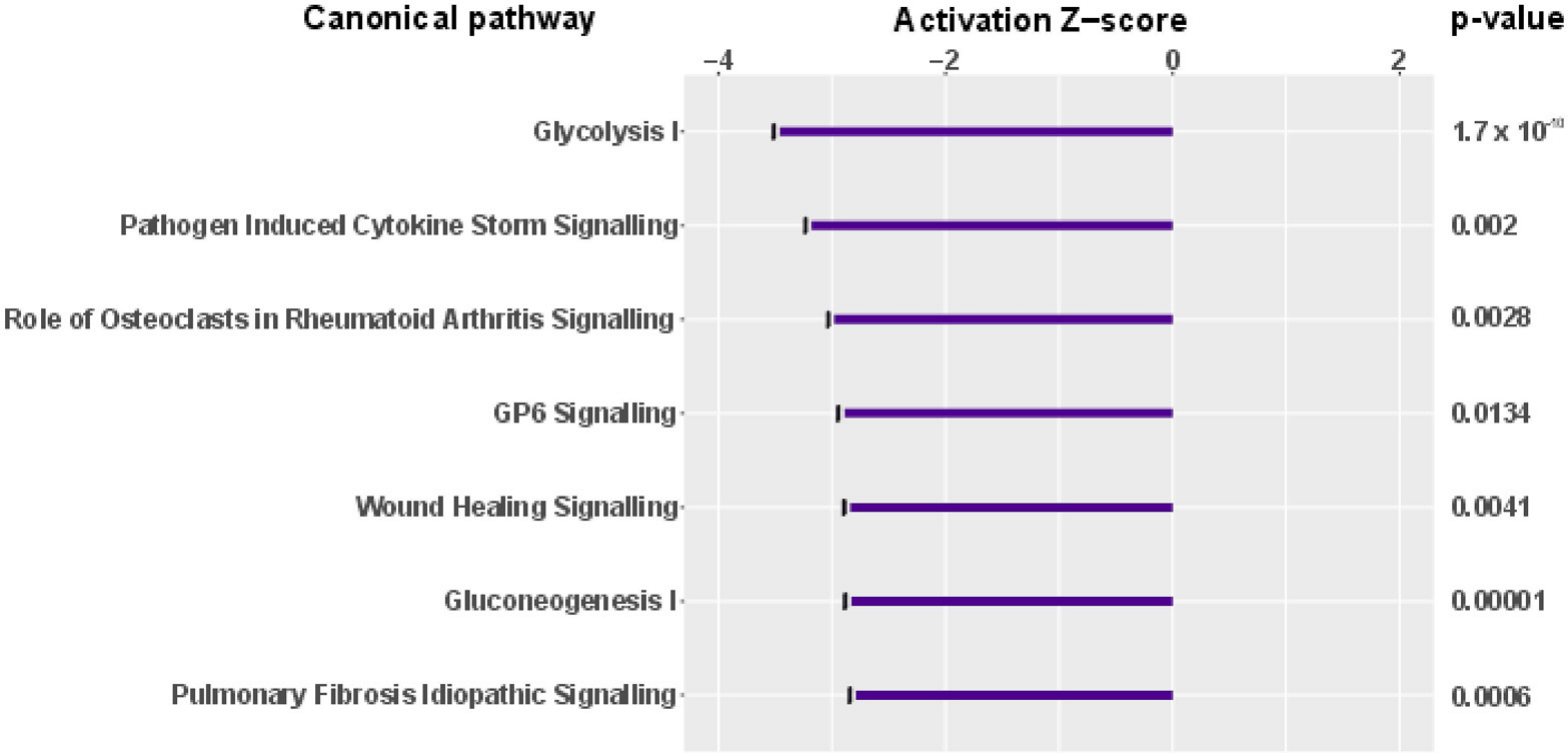
Canonical pathways significantly altered by TRPA1 inhibition predicted by Ingenuity Pathway Analysis. Significant predicted change in pathway activity was |*Z*‐score| > 2.5 and *p* < 0.05. The pathway analysis was conducted with genes differentially expressed by both TRPA1 antagonists in same direction (|FC > 1.5|, *p*‐adj < 0.05).

IPA was conducted on genes that had their expression similarly changed by both TRPA1 antagonists to assess possible biological effects of TRPA1 inhibition on A549 cells under inflammatory conditions. In total, seven ingenuity canonical pathways were predicted to be inhibited by TRPA1 antagonists, and none was activated (|*z*‐score| > 2.5 and *p* < 0.05) (Figure 1). The canonical pathways that were predicted to be inhibited by TRPA1 antagonist in the analysis included pathways relevant for inflammation and fibrosis: *pulmonary fibrosis idiopathic signalling*, *pathogen‐induced cytokine storm signalling*, *wound healing signalling*, *GP6 signalling* and *role of osteoclasts in rheumatoid arthritis signalling*. Two metabolic pathways were also predicted to be inhibited in IPA analysis: *Glycolysis I* and *Gluconeogenesis I*.

**FIGURE 2:**
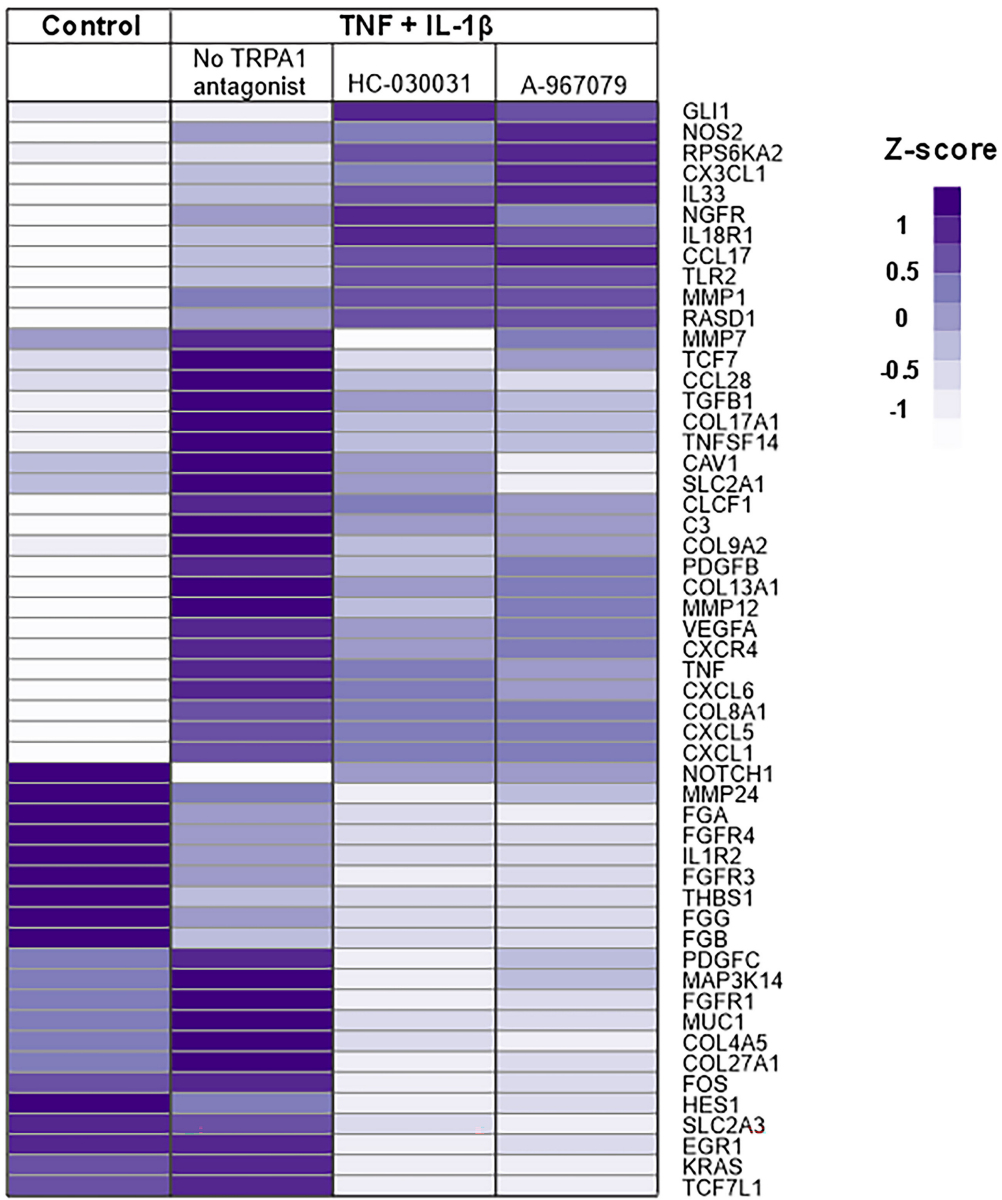
Heatmap of genes included in *pulmonary fibrosis idiopathic signalling*, *wound healing* or *cytokine storm signalling* pathways whose expression was significantly changed by TRPA1 inhibitors. Each column represents mean counts for the treatment group *z*‐standardized row‐wise for each gene. Lighter colour signifies lower expression in relation to other groups within the same row. *n* = 4 samples per treatment.

Because our aim was to investigate the effects of TRPA1 on pulmonary inflammation and fibrosis, we chose three pathways for further investigation: *pulmonary fibrosis idiopathic signalling*, *wound healing signalling* and *pathogen‐induced cytokine storm signalling* pathways. In total, these three pathways contained 53 genes whose expression was altered by the treatment of both TRPA1 antagonists—these are shown as heatmap (Figure 2).

Next, we selected genes included in the pathway *pulmonary fibrosis idiopathic signalling* whose expression was significantly altered by TNF + IL‐1β (|FC| > 1.5, FDR < 0.05), and the effect was prevented by the treatment with the TRPA1 antagonists. There were 13 such genes in total. The expression of 12 of these genes was increased by the inflammatory cytokines and decreased by the TRPA1 antagonists as shown in Table 1. These genes include MMPs, growth factors, collagens and transcription factors (Table 1). One gene was downregulated by TNF + IL‐1β and upregulated by TRPA1 antagonists: NOTCH1 (Notch Receptor 1).

**TABLE 1:** Genes present in the *pulmonary fibrosis idiopathic signalling* pathway that were upregulated by TNF + IL‐1β and the effect was reversed by TRPA1 antagonists; *p* values are FDR adjusted.

		TNF + IL‐1β vs. control		TNF + IL‐1β vs. TRPA1 inhibitor treated			
Gene	Protein name	Fold change (FC)	*p*‐adj	FC (vs. HC‐030031)	*p*‐adj	FC (vs. A‐967079)	*p*‐adj
EGR1	Early growth response protein 1	1.58	0.040894	−8.14	< 1 × 10^−6^	−4.01	0.000034
MMP12	Matrix metalloproteinase 12	588.07	< 1 × 10^−6^	−7.15	< 1 × 10^−6^	−2.97	0.000014
FOS	Protein c‐Fos	1.97	0.031282	−6.27	< 1 × 10^−6^	−5.07	0.000011
MMP7	Matrix metalloproteinase 7	2.91	< 1 × 10^−6^	−5.89	< 1 × 10^−6^	−1.66	< 1 × 10^−6^
COL13A1	Collagen alpha‐1(XIII) chain	51.23	< 1 × 10^−6^	−4.31	< 1 × 10^−6^	−2.49	0.004000
COL9A2	Collagen alpha‐2(IX) chain	8.52	0.000187	−3.18	0.000120	−2.06	0.022000
TCF7	Transcription factor 7	2.79	< 1 × 10^−6^	−3.04	< 1 × 10^−6^	−1.98	< 1 × 10^−6^
PDGFB	Platelet‐derived growth factor subunit B	8.74	< 1 × 10^−6^	−3.02	< 1 × 10^−6^	−1.77	0.000025
COL17A1	Collagen alpha‐1(XVII) chain	2.48	0.000064	−2.17	< 1 × 10^−6^	−2.09	0.000880
CAV1	Caveolin‐1	1.84	< 1 × 10^−6^	−1.82	< 1 × 10^−6^	−2.77	< 1 × 10^−6^
TGFB1	Transforming growth factor beta‐1 proprotein	2.01	< 1 × 10^−6^	−1.79	< 1 × 10^−6^	−1.98	< 1 × 10^−6^
COL8A1	Collagen alpha‐1(VIII) chain	1512.48	< 1 × 10^−6^	−1.53	0.005800	−1.57	0.035000

**FIGURE 3:**
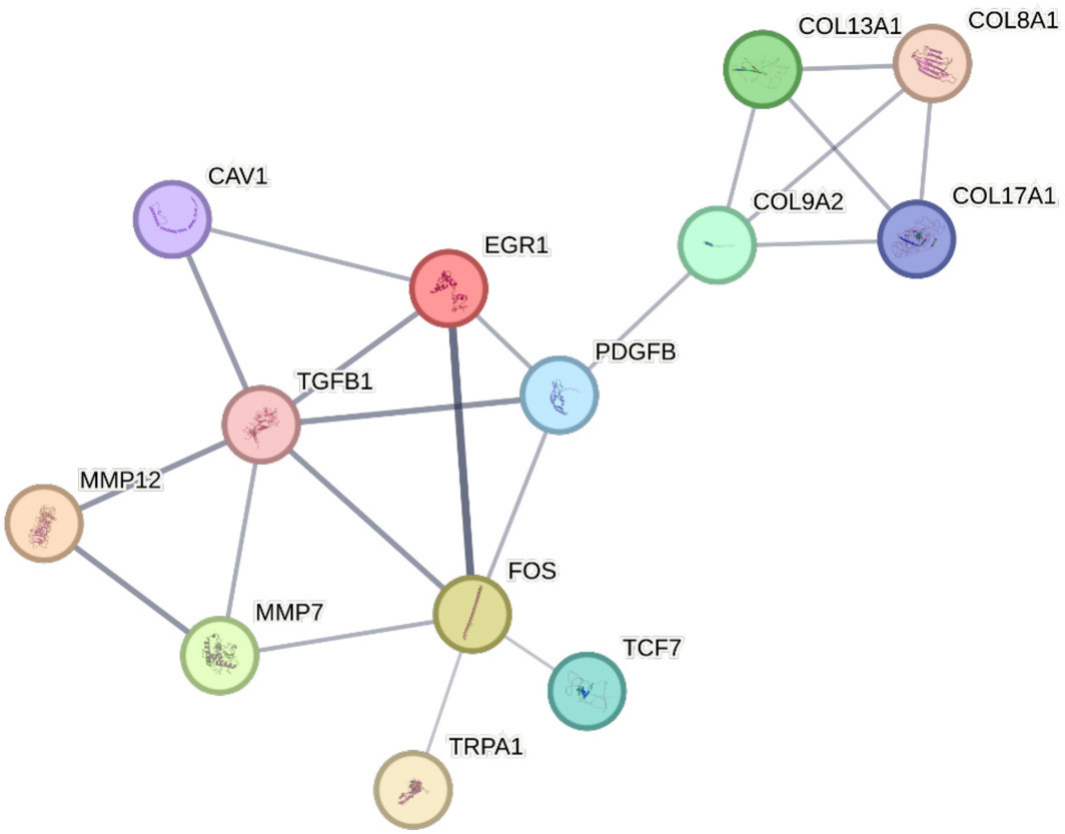
STRING network of proteins in *pulmonary fibrosis idiopathic signalling* pathway, which were upregulated by TNF + IL‐1β and downregulated by the TRPA1 antagonists. Nodes represent proteins, and a vertex is present if there is known or suspected interaction between two proteins. Thickness of a vertex represents strength of the evidence.

To investigate the known interactions between the proteins encoded by the genes in Table 1, we created a STRING network with these factors and TRPA1, which searches for physical and functional associations between given proteins from known databases and through text mining from published articles. The network shows TRPA1 being connected to the other genes through FOS (Figure 3). Downregulated collagens seem to cluster together and associate with TRPA1 through PDGFB and FOS (Figure 3).

### 3.3 Matrix Metalloproteinases MMP‐12 and MMP‐7

Among the genes with the largest amplitude of downregulation by the TRPA1 antagonist HC‐030031 are matrix metalloproteinase 12 (MMP‐12), matrix metalloproteinase 7 (MMP‐7), early growth response 1 (EGR1) and Fos proto‐oncogene (FOS). MMP‐12 and MMP‐7 were chosen for further investigation, as these have been linked to worse prognosis of pulmonary fibrosis (in the case of MMP‐7) [36, 37] and to the pathogenesis of pulmonary fibrosis and emphysema (in the case of MMP‐12) [38, 39]. In the RNA‐seq data, MMP‐12 mRNA expression was barely detectable in untreated control samples, but treatment with TNF and IL‐1β induced an over 500‐fold increase in its expression. MMP‐7 had a higher baseline expression than MMP‐12 and a smaller, but statistically significant, relative increase in expression following TNF and IL‐1β treatment (2.91‐fold) (Table 1).

**FIGURE 4:**
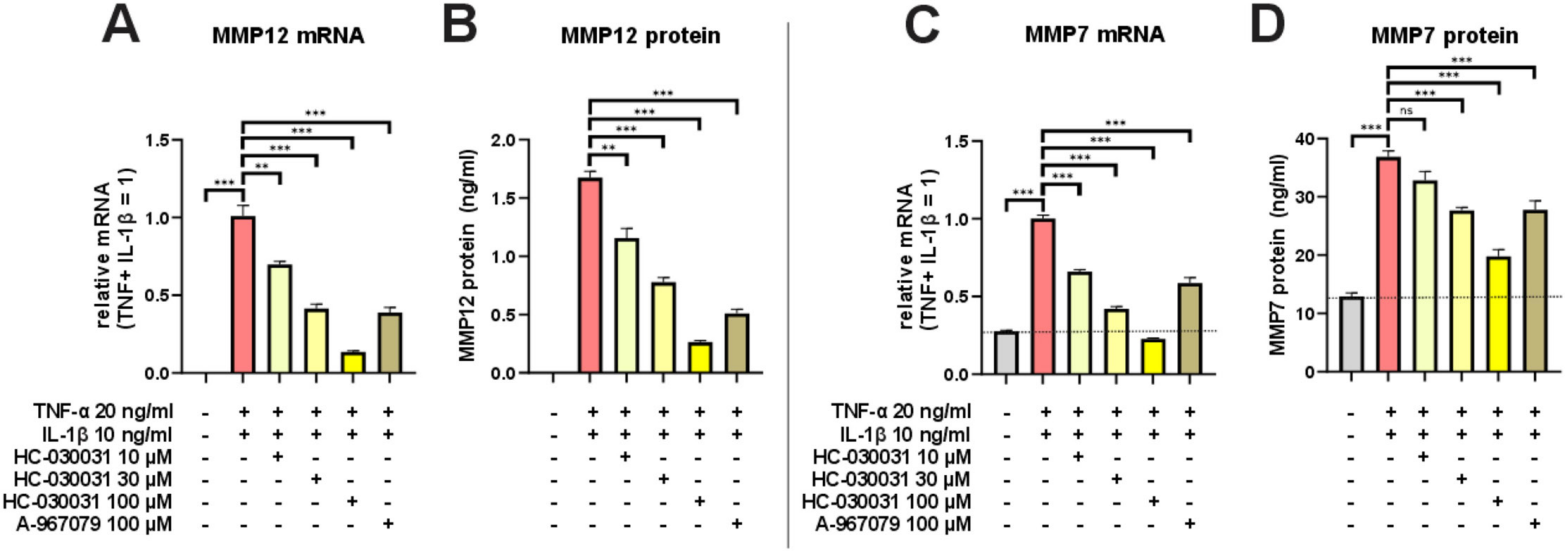
The effect of TRPA1 antagonists on MMP‐12 (A,B) and MMP‐7 (C,D) mRNA and protein expression as measured with RT‐PCR and ELISA, respectively. A549 were treated with TNF (20 ng/mL) and IL‐1β (10 ng/mL) with or without the TRPA1 antagonist HC‐030031 or A‐967079 and cultured for 24 h, after which culture medium was collected and total RNA extracted. In RT‐PCR, RPL‐30 was used as a reference gene, and the relative expression is normalized against TNF and IL‐1β treated cells. Data are presented as mean fold change compared to TNF + IL‐1β treated cells with SEM as error bar. Statistical significance was tested with Welch ANOVA with the Holm–Bonferroni corrected groupwise comparisons. *n* = 6. *p < 0.05, **p < 0.01, ***p < 0.001, ns = not significant.

First, we validated by RT‐PCR the effects of the TRPA1 antagonists on MMP‐12 and MMP‐7 expression that were seen in the RNA‐Seq. Consistently, inflammatory cytokines TNF and IL‐1β induced a considerable increase in their mRNA levels, which was attenuated by the TRPA1 antagonists HC‐030031 and A‐967079 (Figure 4). Next, we assessed whether the effects of the TRPA1 antagonists also translate to protein levels. For this, ELISA was used to measure protein levels in cell culture media. Although some baseline secretion of MMP‐7 was seen in cells without inflammatory stimulation, MMP‐12 was not detected in the medium of untreated cells (Figure 4). Inflammatory stimulus with TNF and IL‐1β massively increased the secreted protein levels of both MMP‐7 and MMP‐12. This increase in protein levels was attenuated by the TRPA1 antagonists in a dose‐dependent manner (Figure 4).

**FIGURE 5:**
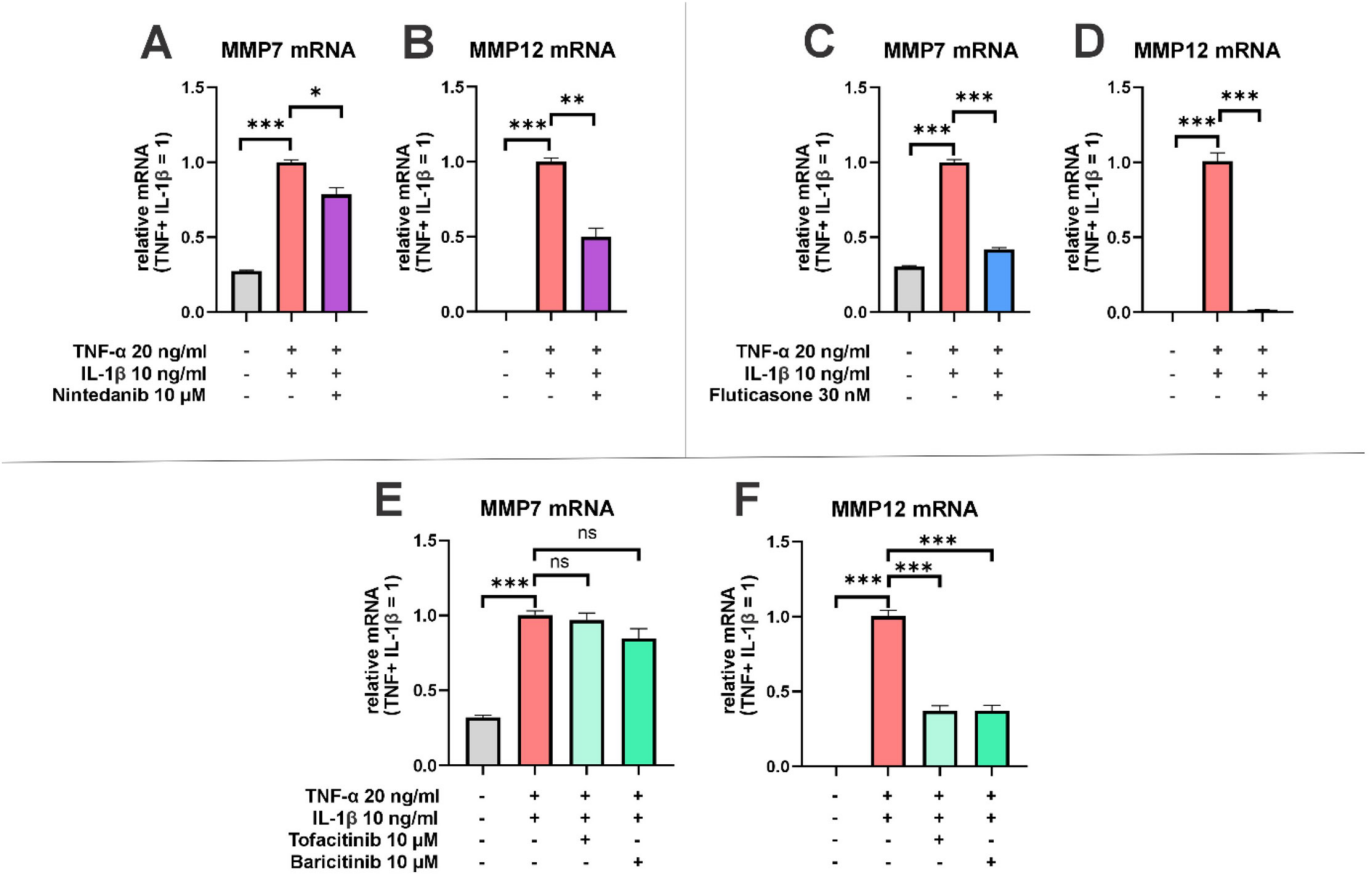
The effects of anti‐inflammatory or anti‐fibrotic drugs (fluticasone, nintedanib, JAK inhibitors baricitinib and tofacitinib) on MMP‐7 and MMP‐12 expression as measured with RT‐PCR. A549 cells were treated with indicated combinations of TNF (20 ng/mL) and IL‐1β (10 ng/mL) and drugs nintedanib 10 μM (A,B), fluticasone 30 nM (C,D), tofacitinib 10 μM or baricitinib 10 μM (E,F). Cells were cultured for 24 h, after which total RNA was extracted. RPL‐30 was used as a reference gene, and the relative expression is normalized against TNF and IL‐1β treated cells. Data are presented as mean fold change compared to TNF + IL‐1β‐treated cells with SEM as error bar. Statistical significance was tested with Welch ANOVA with the Holm–Bonferroni corrected groupwise comparisons. *n* = 6. *p < 0.05, **p < 0.01, ***p < 0.001, ns = not significant.

Next, we investigated how the effects of the TRPA1 antagonists compare to some clinically used antifibrotic or anti‐inflammatory drugs. The glucocorticoid fluticasone propionate almost completely reversed the upregulation of MMP‐12 and MMP‐7 after treatment with TNF + IL‐1β (Figure 5). Nintedanib, an antifibrotic drug with indication for IPF, also decreased the expression of MMP‐12 and MMP‐7 albeit the effect was less pronounced than with the TRPA1 antagonists (Figure 5). The JAK inhibitors baricitinib and tofacitinib decreased the expression of MMP‐12 but had no significant effect on MMP‐7 mRNA levels (Figure 5).

## 4 Discussion

This study provides evidence that TRPA1 is involved in the regulation of the expression of more than 900 genes in pulmonary epithelial cells under inflammatory conditions. Pathway analysis predicts that the genes regulated by TRPA1 are involved in processes such as fibrosis, inflammation and metabolism. Among the genes most strongly downregulated by TRPA1 antagonists are MMP‐12 and MMP‐7, which are both linked to the development of fibrosis. These findings extend the previous understanding on the role of TRPA1 in lung inflammation and fibrosis [5, 21, 24].

Previous studies have reported that the pro‐inflammatory cytokines TNF and IL‐1β increase the expression of TRPA1 in lung epithelial cells and chondrocytes [5, 7]. The present RNA‐sequencing results showed a similar increase, confirming the previous findings on inflammatory regulation of TRPA1 expression.

IPA predicted the suppression of fibrosis‐associated transcriptional programs by TRPA1 antagonists. In *pulmonary fibrosis idiopathic signalling* pathway, attention was directed particularly towards MMP‐12 and MMP‐7, which were highly upregulated by TNF and IL‐1β, and this effect was greatly attenuated by both of the investigated TRPA1 antagonists. Furthermore, previous research shows an important role for these MMPs in fibrosis and pulmonary inflammatory diseases [37, 38, 39, 40]. In our RNA‐seq data (Table 1), the expression of MMP‐12 was increased over 500‐fold by TNF and IL‐1β treatment, and this increase was significantly attenuated by TRPA1 antagonists.

MMP‐12, also known as macrophage elastase, was first cloned from human alveolar macrophages [41] and has been shown to be secreted also by lung epithelial cells [42]. Classically, MMP‐12 is known to degrade extracellular matrix (ECM) components, most notably elastin and some collagens, and thus, it plays a role in tissue remodelling [43]. Contemporary research also links MMP‐12 to the regulation of inflammation, as MMP‐12 has been shown to modulate the secretion and activity of cytokines such as interferons α and γ [44, 45]. MMP‐12 has been shown to be a crucial factor in the pathogenesis of fibrosis in multiple disease models such as Fas‐induced lung fibrosis [38], silicosis [39] and subretinal fibrosis [46]. MMP‐12 promoter region polymorphism that causes increased activator protein‐1 (AP‐1) binding activity and increased MMP‐12 expression (rs2776109 A/G) is associated with a higher risk of pulmonary fibrosis and systemic sclerosis [47]. Thus, based on research of MMP‐12 in fibrotic disease models, the downregulation of MMP‐12 expression is likely to be beneficial in preventing the progression of fibrosis. MMP‐12 inhibitors in the treatment of asthma and COPD have also entered Phase I and II clinical trials [48, 49].

MMP‐7, also known as matrilysin 1, is produced in lungs by macrophages and epithelial cells in patients with IPF [50]. MMP‐7 is a regulator of lung inflammation after epithelial injury [40], and it is positively correlated with worse prognosis in patients with IPF [36, 37, 51]. MMP‐7 has also been studied as a biomarker of fibrosis in non‐alcoholic fatty liver disease patients [52]. On top of the role of MMP‐7 as a biomarker, it has also been clearly associated with the pathogenesis of fibrotic diseases. MMP‐7‐deficient mice were protected from the development of fibrosis in the bleomycin‐induced pulmonary fibrosis model [53]. In a newer study, development of bleomycin‐induced fibrosis in the mouse model was alleviated by *Tylophora indica* extract which contains compounds shown to be potent MMP‐7 inhibitors [54]. Thus, based on previous research on MMP‐7 in fibrosis, the downregulation of MMP‐7 expression by TRPA1 antagonists is likely to protect from fibrosis. The ability of TRPA1 antagonists to downregulate both MMP‐7 and MMP‐12 on mRNA and protein level presents the ion channel as an interesting drug target in lung inflammation and fibrosis.

There are several plausible mechanisms through which TRPA1 might regulate the expression of MMP‐12 and MMP‐7. MMP‐12 promoter has a binding site for AP‐1 that seems to be preferred by Jun‐Fos heterodimers [55]. Our RNA‐seq data show a strong downregulation of FOS gene (encoding for c‐Fos protein) by TRPA1 antagonists (Table 1). As c‐Fos protein is part of the AP‐1 transcription factor heterodimer, this downregulation may lead to reduced AP‐1‐dependent activation of MMP‐12 and MMP‐7 gene transcription. This hypothesis is in line with the present STRING network analysis, which suggests that TRPA1 is connected to MMP‐12 and MMP‐7 through FOS (Figure 3). It should be noted, however, that the STRING network is built by data mining from different data sets and databases and thus offers only suggestive, not confirmatory, links. This hypothesis should be further investigated in follow‐up studies aimed at studying the causative role of TRPA1 on AP‐1‐mediated transcription.

Another mechanism by which TRPA1 may regulate gene expression is through protein kinase C. TRPA1 activation has been suggested to increase PLC and PKC signalling, as inhibiting PLC or PKC alleviates hyperalgesia caused by TRPA1 agonists [56]. PKC activation has been shown to increase MMP‐7 expression [57], and it seems to be needed also for MMP‐12 upregulation, as PKC‐deficient mice had lower expression of MMP‐12 after asbestos exposure than WT mice [58]. TRPA1 itself is also regulated by PLC and PKC [12, 13]. Thus, PKC signalling might be a possible pathway through which TRPA1 modulates MMP‐7 and MMP‐12 expressions.

The effect of TRPA1 antagonists on MMP‐7 and MMP‐12 expression was compared to other drugs used for the treatment of pulmonary diseases (Figure 3). Nintedanib, an antifibrotic drug that is known to inhibit multiple tyrosine kinases, was also found to suppress MMP‐7 and MMP‐12 expression. A similar effect, although somewhat more pronounced, was seen with fluticasone propionate, a glucocorticoid used in the treatment of asthma [59] and, in some cases, COPD [60]. Interestingly, dexamethasone, another glucocorticoid, has been shown to downregulate TRPA1 expression post‐transcriptionally by increasing the rate of TRPA1 mRNA degradation [5]. Therefore, it is possible that the inhibitory effect of fluticasone on MMP‐12 and MMP‐7 expression in A549 cells is mediated, at least partly, by reduced TRPA1 expression.

The JAK1/2 inhibitor baricitinib [61] and the JAK1/3 inhibitor tofacitinib [61] diminished the effect of TNF and IL‐1β on MMP‐12 expression but not on MMP‐7. This suggests that the expression of these MMPs is partly regulated by different pathways. The fact that TRPA1 antagonists managed to decrease the expression of both MMP‐12 and MMP‐7 implies that TRPA1 has a wider effect in inflammation, which is not linked to JAK/STAT signalling. It should be noted, however, that neither TNF nor IL‐1β directly activates JAK signalling, which means that the effect of the JAK pathway on MMP‐12 expression is likely an indirect effect.

*Pulmonary fibrosis idiopathic signalling* and *wound healing signalling* pathways have significant overlap between genes. This is not surprising, as both fibrosis and wound healing involve similar processes, including differentiation of fibroblasts into myofibroblasts capable of contracting and secreting collagens and other ECM components [62]. Fibrosis can be seen as a pathological wound healing process where remodelling of connective tissue is not terminated properly, thus leading to excessive scar tissue formation and loss of function in the affected organ [62, 63]. A single‐cell RNA‐sequencing study on epithelial cells in IPF patients showed enrichment in ‘response to wounding’ process (and others involved in ECM remodelling) by genes induced by IPF [64]. TRPA1 has also been shown to play a role in epithelial cell injury caused by cigarette smoke [65], and sustained epithelial injury is one of the drivers of pulmonary fibrosis [66]. Thus, this study, combined with previous knowledge about the role of epithelium in fibrosis, implies that epithelial TRPA1 has a role in the development of pulmonary fibrosis.

The development of fibrosis is an interplay of multiple cell types, particularly epithelial cells, fibroblasts and macrophages. In macrophages, TRPA1 is shown to drive macrophage polarization towards the profibrotic M2 phenotype, and the knock‐out of TRPA1 is protective against scleroderma‐type skin fibrosis [67]. In fibroblasts, TRPA1 may have a tissue‐specific effect: In lung fibroblasts, the activation of TRPA1 was reported to inhibit fibroblast‐to‐myofibroblast transformation [68] and reduce the survival of myofibroblasts [69]. In contrast, in ocular and cardiac fibroblasts, the activation of TRPA1 was shown to drive profibrotic changes that were alleviated by TRPA1 inhibition [70, 71]. In disease models, such as idiopathic pulmonary fibrosis model and skin fibrosis, the net effect of TRPA1 seems to be profibrotic, as TRPA1 knock‐out has been shown to protect from fibrotic changes [67, 72].

In lung epithelial cells, the possible anti‐ or profibrotic roles of TRPA1 have not been previously investigated. We show here that in human lung epithelial cells, the inhibition of TRPA1 downregulates fibrotic genes and pathways at transcriptome level and decreases the secretion of profibrotic MMP7 and MMP12. Thus, this study supports the profibrotic role of TRPA1 seen in animal models [67, 72] and suggests the involvement of epithelial TRPA1 in lung fibrosis.

Interestingly, IPA also predicted suppression of glycolysis‐associated transcriptional programmes (Figure 1) by TRPA1 inhibitors. Previous research has shown that pro‐inflammatory stimulus can drive cellular metabolism towards glycolysis [73], and our results from RNA‐seq and IPA suggest that TRPA1 inhibitors can reverse this change (Figure 1). However, we cannot rule out the possibility that the baseline rate of glycolysis in the present experimental model was already somewhat elevated, which has been reported in in vitro cell cultures and tumour cells [74, 75]. In either case, TRPA1 antagonists seem to downregulate transcription of genes associated with glycolysis. The possible effect of TRPA1 on glycolysis and immunometabolism merits further investigation.

The present study was conducted with A549 cell line, which is a well‐established experimental model for pulmonary epithelial cells [76]. However, it is an immortalized cell line and thus might not fully recapitulate primary epithelial biology. Also, as the cells are from a single biological source, the results may not be as readily generalizable as those obtained using primary cells. However, using A549 cells enables our reductionist approach to study the mechanistic effect of TRPA1 inhibition on lung epithelial cells, avoiding confounding factors present in tissue samples or primary cell cultures. Further studies are needed to assess the translational relevance of the present findings in primary cell cultures and pathophysiological disease models.

### 4.1 Conclusion

TRPA1 antagonists were found to change the expression of over 900 genes and predicted to inhibit the activity of multiple biological canonical pathways associated with inflammation, fibrosis and metabolism in lung epithelial cells under inflammatory conditions. Among the genes whose expression was most significantly increased by inflammatory stimulus and downregulated by TRPA1 antagonists are MMP‐12 and MMP‐7, which have a major role in the pathogenesis of lung inflammation and fibrosis. These results suggest that TRPA1 is involved in the regulation of lung inflammation and fibrosis and support further investigation of TRPA1 in fibrosis models.

## Funding

This work was supported by the Research Council of Finland; the Tampere Tuberculosis Foundation, Finland; and the Competitive Research Funding of Tampere University Hospital (VTR Funding).

## Conflicts of Interest

The authors declare no conflicts of interest.

## Supporting information

TRPA1 regulates fibrosis‐associated transcriptional pathways in human lung epithelial cells Leevi Halonen, Samu Luostarinen, Ida Valjus, Julia Vistbacka, Antti Pemmari, Eeva Moilanen The Immunology Research Group, Faculty of Medicine and Health Technology, Tampere University and Tampere University Hospital, Wellbeing Services County of Pirkanmaa, Tampere, Finland.

## Data Availability

The data that support the findings of this study are available from the corresponding author upon reasonable request.
