# Supplementary material for: TRPA1 Regulates Fibrosis‐Associated Transcriptional Pathways in Human Lung Epithelial Cells
Source: Basic Clin Pharmacol Toxicol. 2026 Aug 5;139(3):e70276. doi: 10.1111/bcpt.70276 (PMC13443396; doi:10.1111/bcpt.70276)
Supplement: Supplementary file 1 [file BCPT-139-0-s001.pdf]

# Supplementary Data: TRPA1 regulates fibrosis-associated transcriptional pathways in human lung epithelial cells

Leevi Halonen, Samu Luostarinen, Ida Valjus, Julia Vistbacka, Antti Pemmari, Eeva Moilanen
The Immunology Research Group, Faculty of Medicine and Health Technology, Tampere University and Tampere University Hospital, Wellbeing Services County of Pirkanmaa, Tampere, Finland

## RNA-Seq Quality Control metrics

### 1. Read quality table

| sample | raw_reads | raw_bases | clean_reads | lean_base | error_rate | Q20 | Q30 | GC_pct |
|---|---|---|---|---|---|---|---|---|
| Control 1 | 43721778 | 6.56G | 43281390 | 6.49G | 0.03 | 97.47 | 92.91 | 49.4 |
| Control 2 | 53022260 | 7.95G | 52478402 | 7.87G | 0.03 | 97.62 | 93.23 | 49.87 |
| Control 3 | 43758794 | 6.56G | 43371276 | 6.51G | 0.03 | 97.37 | 92.7 | 49.43 |
| Control 4 | 50445572 | 7.57G | 49806996 | 7.47G | 0.03 | 97.27 | 92.56 | 51.49 |
| TNF + IL-1b 1 | 49640014 | 7.45G | 48957256 | 7.34G | 0.03 | 97.46 | 92.96 | 51.35 |
| TNF + IL-1b 2 | 45767408 | 6.87G | 45087018 | 6.76G | 0.03 | 97.54 | 93.15 | 51.65 |
| TNF + IL-1b 3 | 48511948 | 7.28G | 47961516 | 7.19G | 0.03 | 97.48 | 93.1 | 50.81 |
| TNF + IL-1b 4 | 48108742 | 7.22G | 47531200 | 7.13G | 0.03 | 97.54 | 93.11 | 50.75 |
| HC030031 1 | 53619238 | 8.04G | 53029526 | 7.95G | 0.03 | 97.47 | 93.07 | 50.72 |
| HC030031 2 | 42534038 | 6.38G | 42071670 | 6.31G | 0.03 | 97.44 | 92.93 | 50.84 |
| HC030031 3 | 53831616 | 8.07G | 53155560 | 7.97G | 0.03 | 97.44 | 92.98 | 50.79 |
| HC030031 4 | 68979644 | 10.35G | 68176912 | 10.23G | 0.03 | 97.51 | 93.12 | 50.94 |
| A967079 1 | 51326424 | 7.7G | 50752812 | 7.61G | 0.03 | 97.46 | 92.93 | 49.99 |
| A967079 2 | 40445182 | 6.07G | 40108392 | 6.02G | 0.03 | 97.41 | 92.87 | 50.51 |
| A967079 3 | 41465672 | 6.22G | 41150620 | 6.17G | 0.03 | 97.47 | 93.03 | 50.61 |
| A967079 4 | 40060506 | 6.01G | 39777876 | 5.97G | 0.03 | 97.29 | 92.61 | 50.68 |

*Table S1. RNA-seq read quality table*

**raw_reads** and **raw_bases**: read counts and number of bases before filtering for quality
**clean reads** and **clean_bases:** read counts and number of bases after filtering away low-quality data (G = 10^9)
**error_rate**: error rate for whole sequencing
**Q20** and **Q30**: percentage of read bases with phred value >20 and >30
**GC_pct**: percentage of C and G bases from all read bases

### 2. MA-plots:

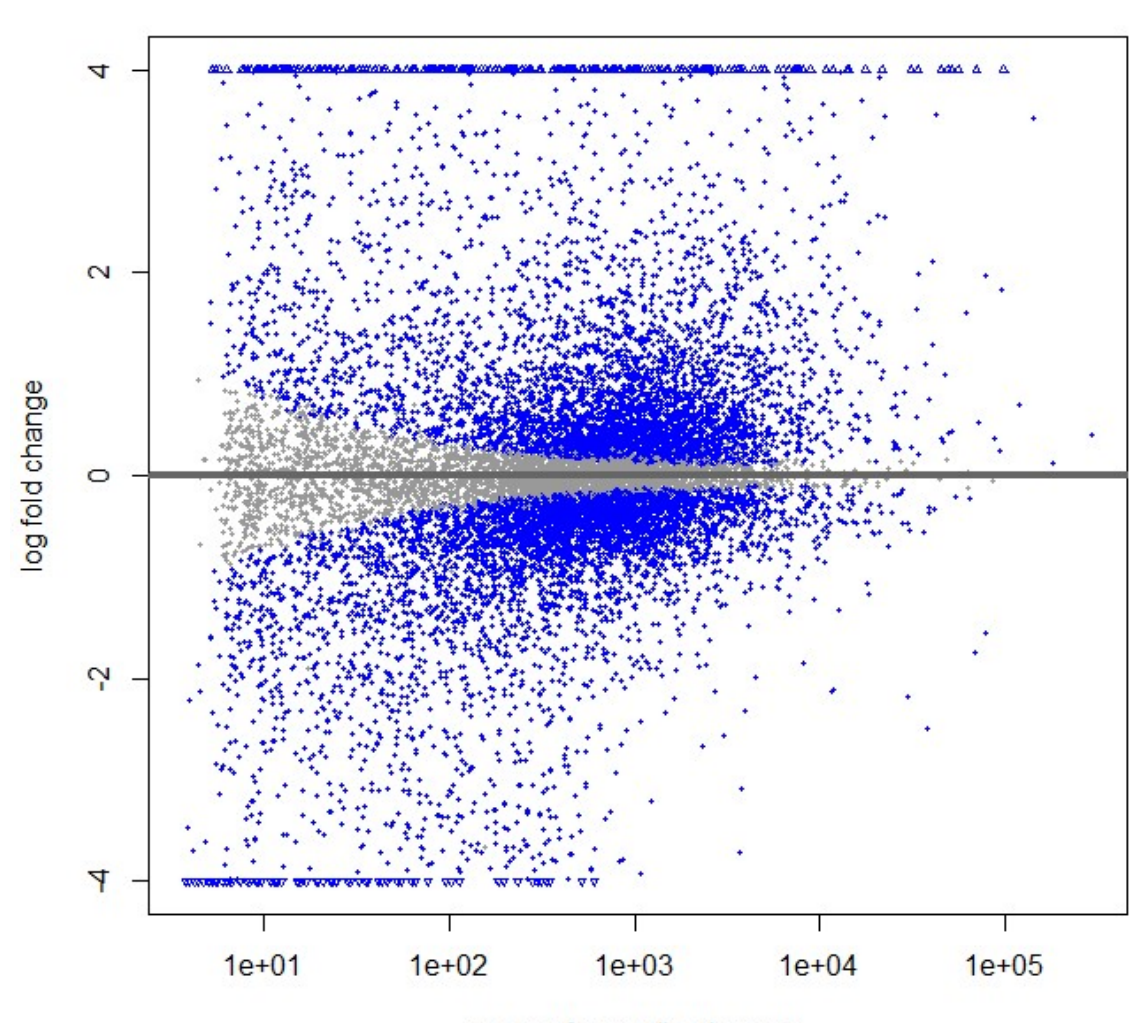


*Figure S1. MA-plot of control vs TNF 20 ng/ml + IL-1b 10 ng/ml*

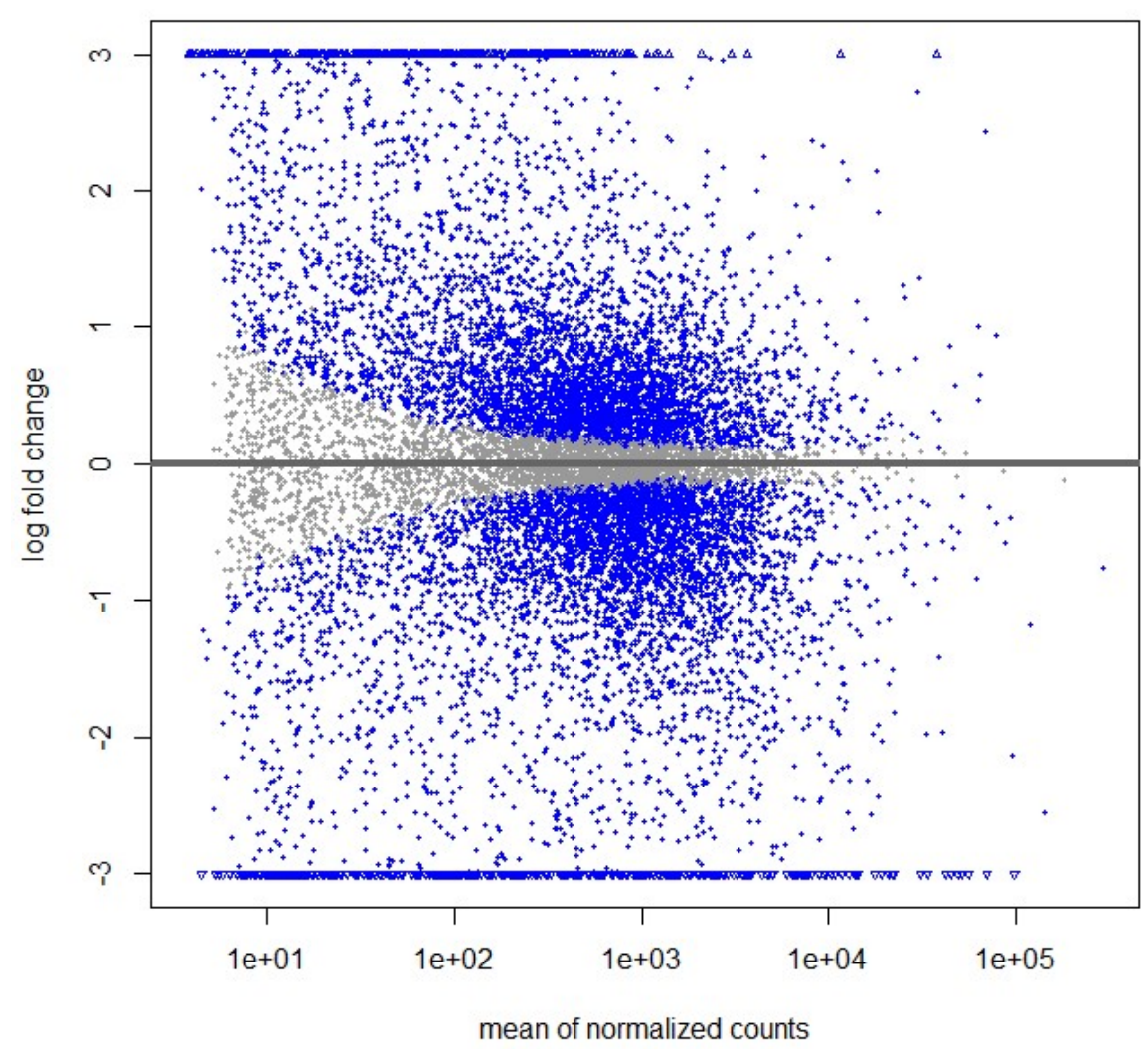


*Figure S2. MA-plot of TNF 20 ng/ml + IL-1b 10 ng/ml + HC030031 100 µM vs TNF 20 ng/ml + IL-1b 10 ng/ml*

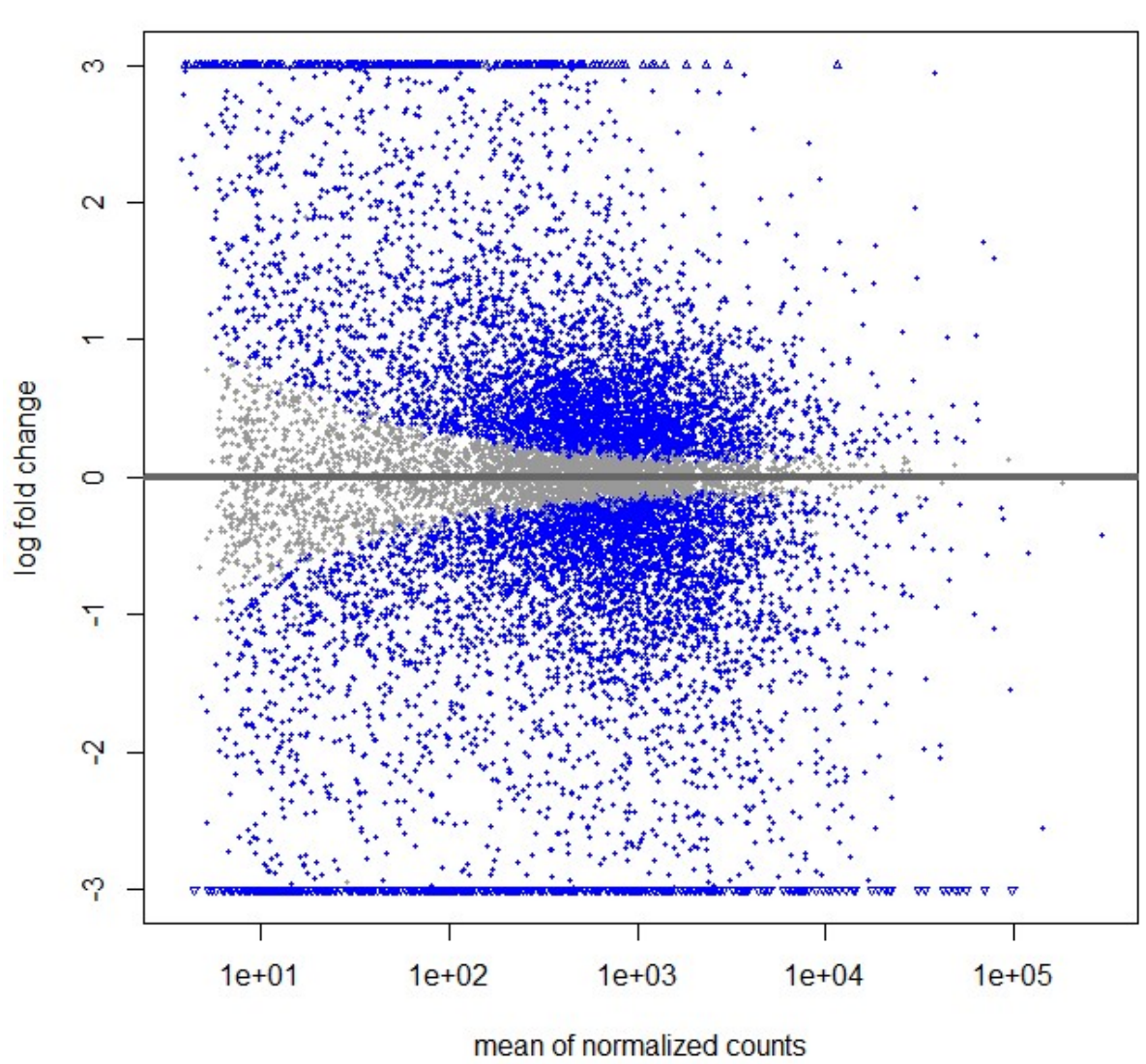


*Figure S3. MA-plot of TNF 20 ng/ml + IL-1b 10 ng/ml + A967079 100 µM vs TNF 20 ng/ml + IL-1b 10 ng/ml*

## 3. PCA-plot:

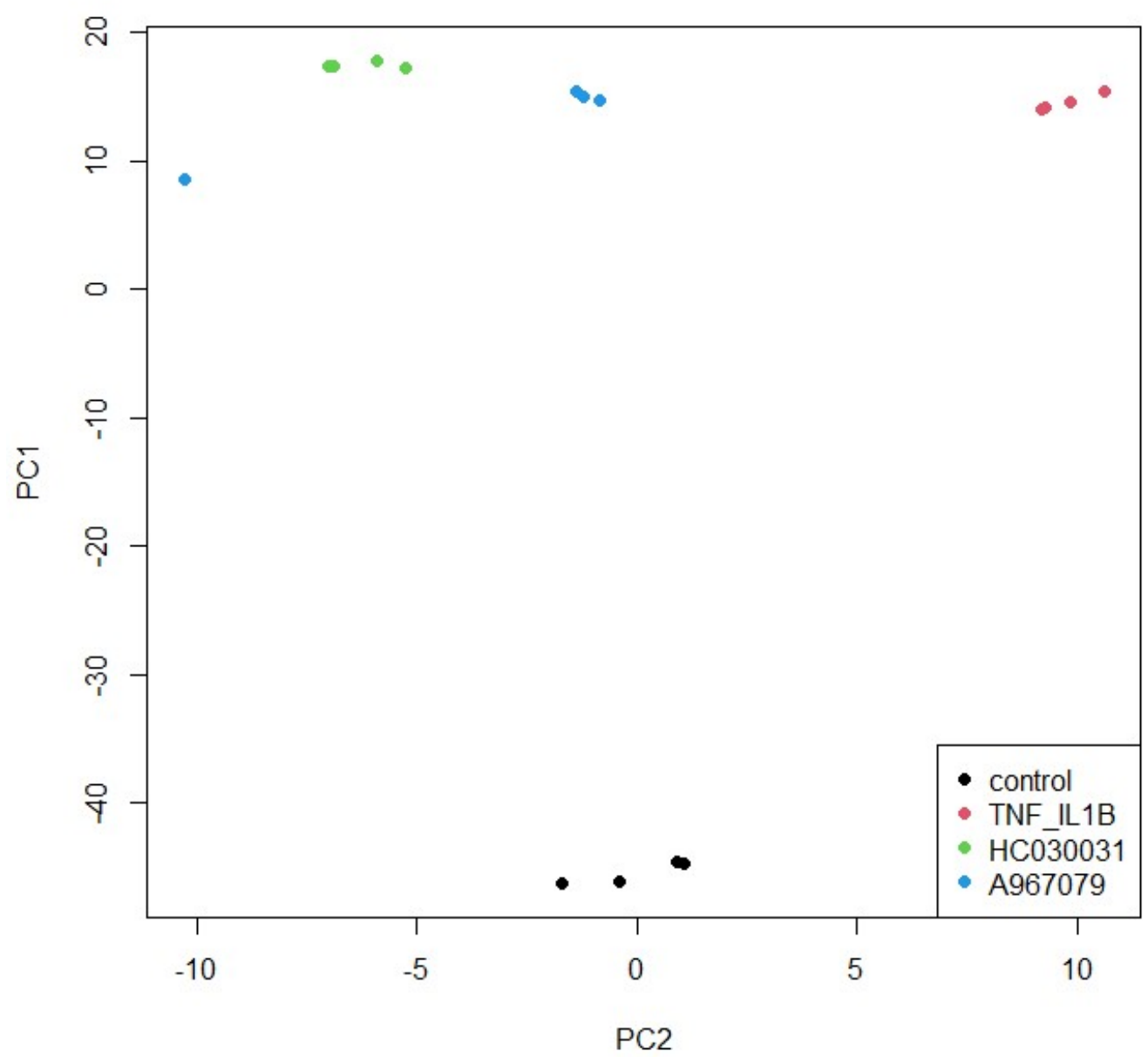


*Figure S4.PCA-plot of two most impactful principal components across the samples used in our experiment*

## 4. Library complexity and gene detection by cutoff across all samples in our experiment

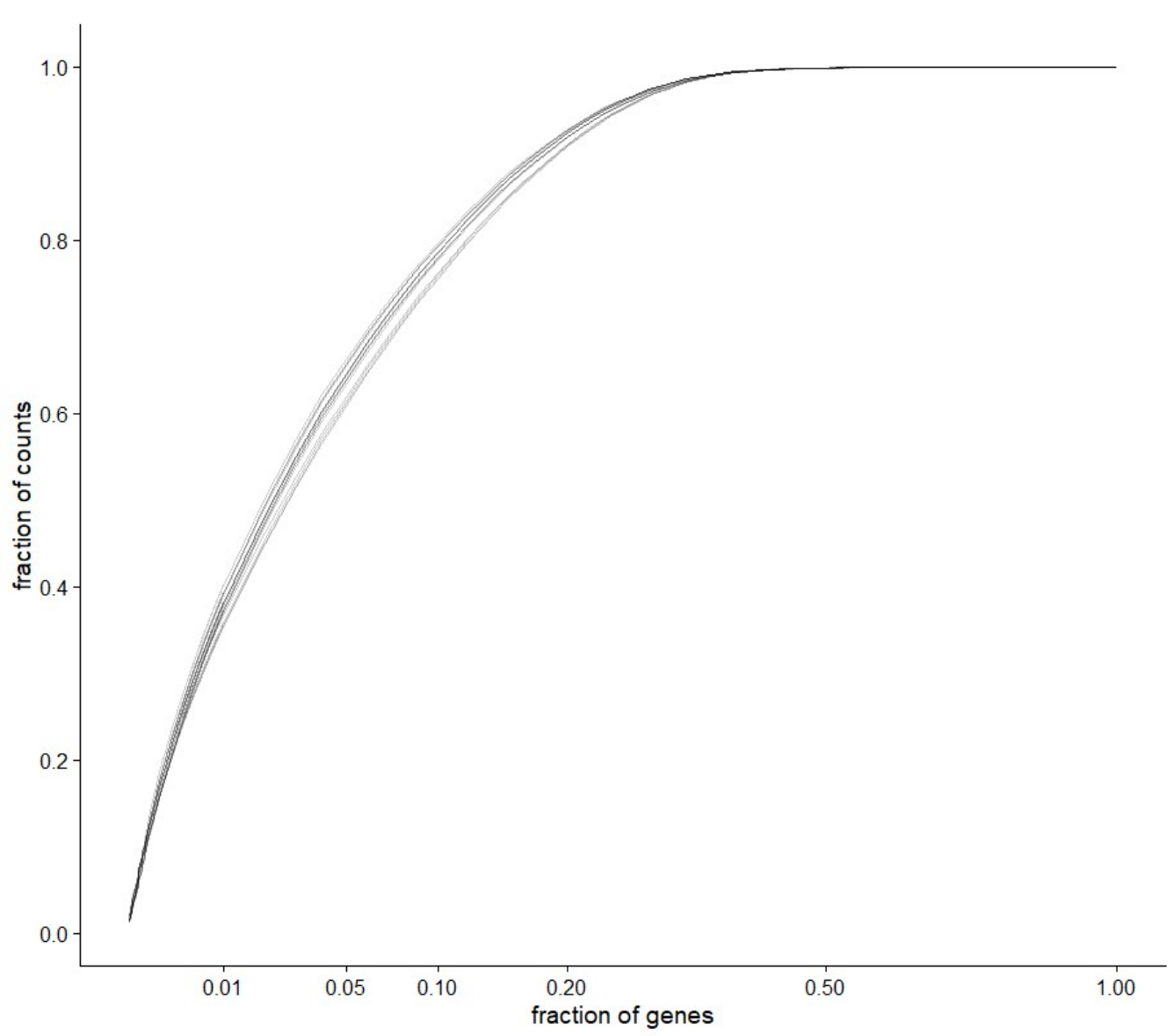


*Figure S5. Library complexity for samples used in the experiment*

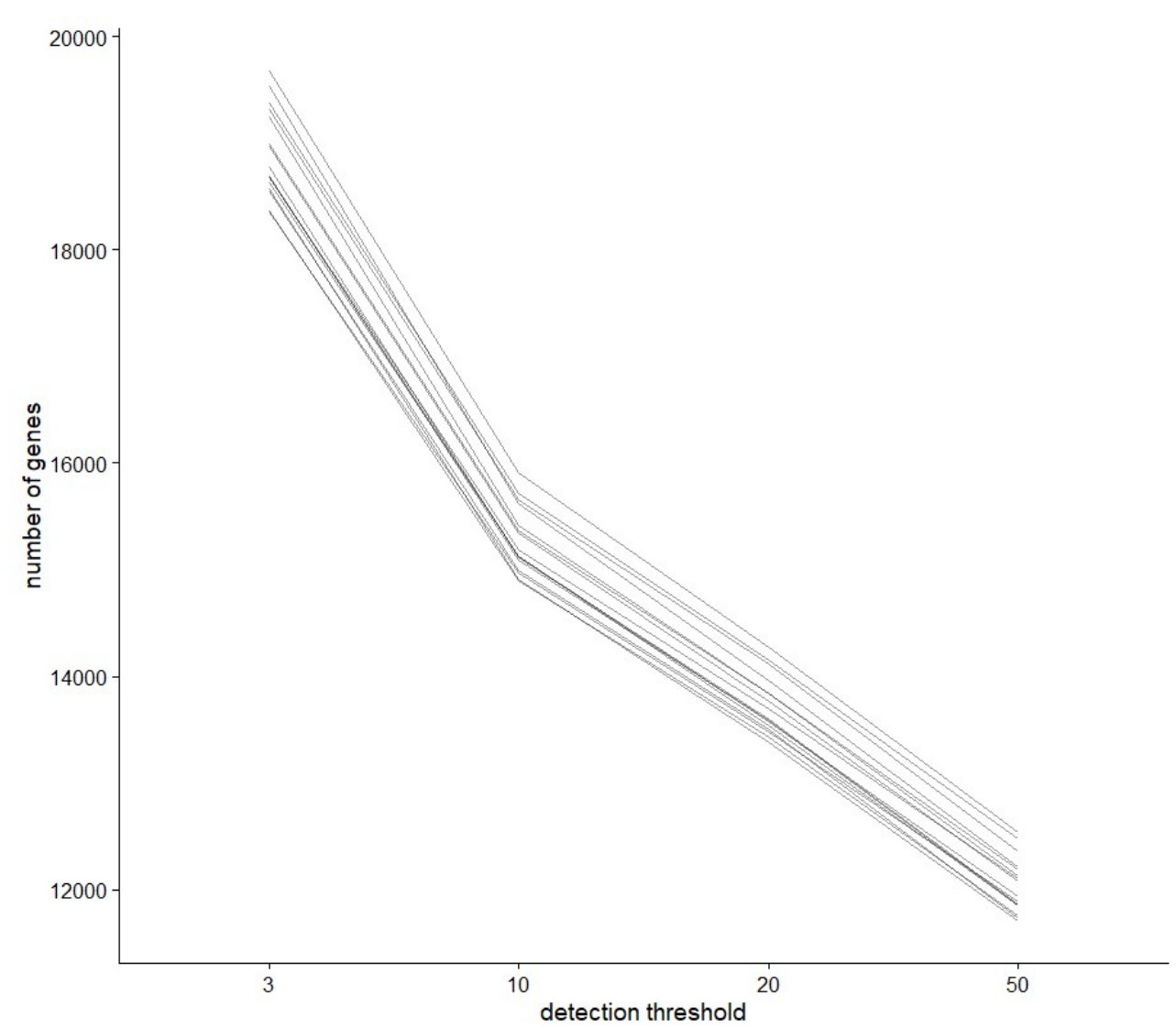


*Figure S6. Detected genes per count cutoff across the samples used in the experiment*
